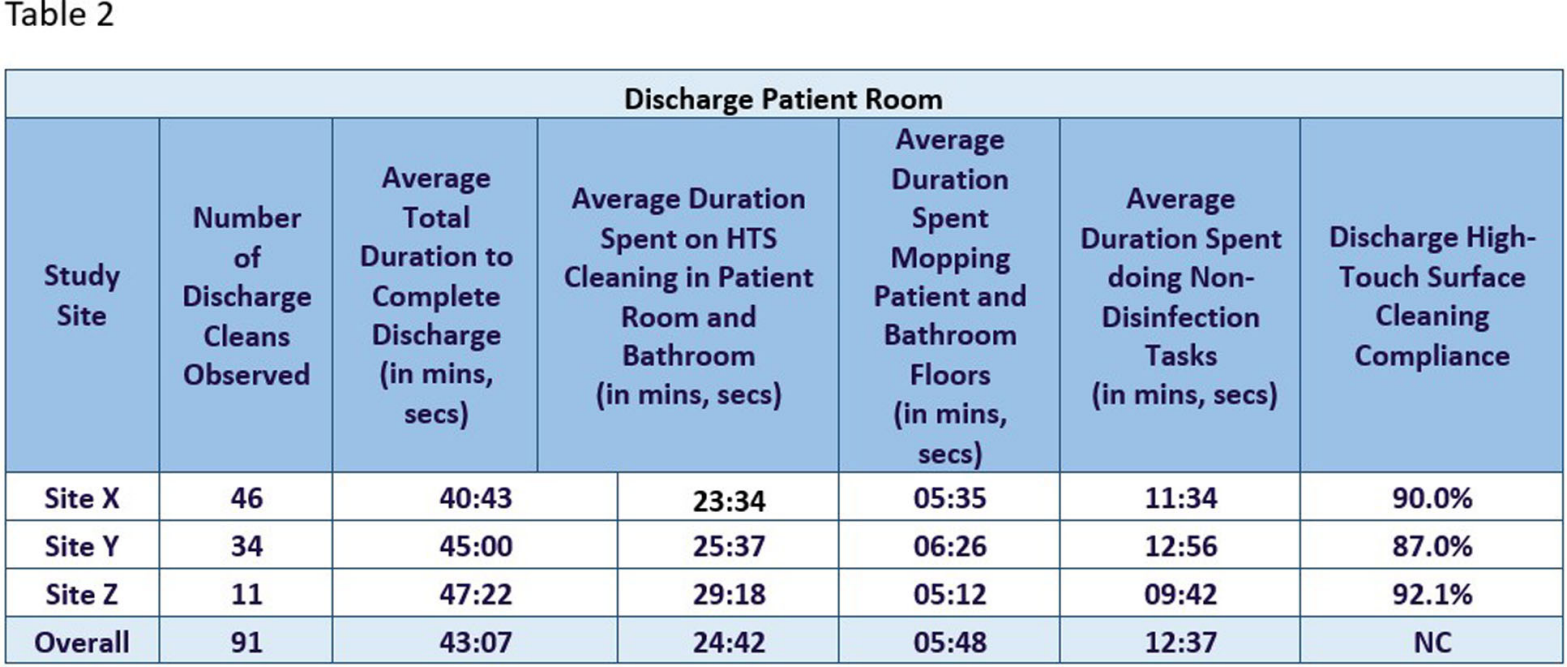# 252 Developing a Measles Flagging System within the Electronic Health Record

**DOI:** 10.1017/ash.2026.10621

**Published:** 2026-06-23

**Authors:** Rebecca Battjes, Peter Teska, Vydia Nankoosingh

**Affiliations:** 1 Diversey - A Solenis Company; 2 Diversey a Solenis company; 3 Diversey- A Solenis Company

## Abstract

**Background:** Environmental hygiene (EH) plays a critical role in reducing healthcare-associated infection (HAI) risk, yet significant variability persists in hospital patient room cleaning and disinfection. While discharge cleaning compliance is frequently studied, real-world practices during daily occupied patient room cleaning remain poorly characterized. Furthermore, the time to complete Environmental Services (EVS) tasks is rarely reported in published literature, despite being linked to budget, facility throughput, and patient and employee satisfaction. Researchers conducted a multi-hospital, observational study about environmental cleaning processes. **Methods:** Across 3 large (<500 beds) teaching hospitals in 3 distinct US regions, researchers shadowed EVS technicians during daily and discharge patient room cleaning across diverse ward types. Direct practice observations documented total turnover time, time to complete tasks such as cleaning the patient and bathroom room surfaces, floor mopping and non-disinfection tasks using a standardized data collection tool (Figure 1). Cleaning compliance was also measured via visual assessment, defined as wiping a surface with a disinfectant wipe or cloth. **Results:** A total of 638 daily and 91 discharge cleans were evaluated. The average occupied daily room turnover time was 9 minutes. However, square footage, isolation needs, in-room equipment and EVS expectations varied across unit types and individual facilities. For daily occupied room cleaning, on average, more time was spent on non-disinfection tasks like removing waste, refilling consumables and patient engagement (see Table 1). High-touch surface (HTS) cleaning compliance during daily cleans was low (38–48%), whereas floor mopping occurred in <90% of rooms. Site Z spent approximately twice the average time performing a daily room clean compared to Sites X and Y, however, did not achieve greater than 50% HTS cleaning compliance. Discharge cleaning demonstrated markedly higher HTS compliance (87–92%) and longer average duration (Table 2). **Conclusion:** To date, EH research has been focused on cleaning compliance measured by fluorescent marking, ATP swabbing and/or environmental culturing. Less research, however, is available regarding daily occupied patient room cleaning and the time and resources needed to do it successfully. Our research revealed that EVS technicians across 3 large hospitals spent an average of 9 minutes on daily room cleaning and only a third of that time spent using a disinfectant. Many healthcare disinfectants used by EVS have a contact time of 5 minutes or more. Healthcare leadership should have realistic expectations of what can be accomplished in budgeted cleaning time and the impact of time on disinfectant efficacy, regulatory compliance and infection prevention.

**Figure:**
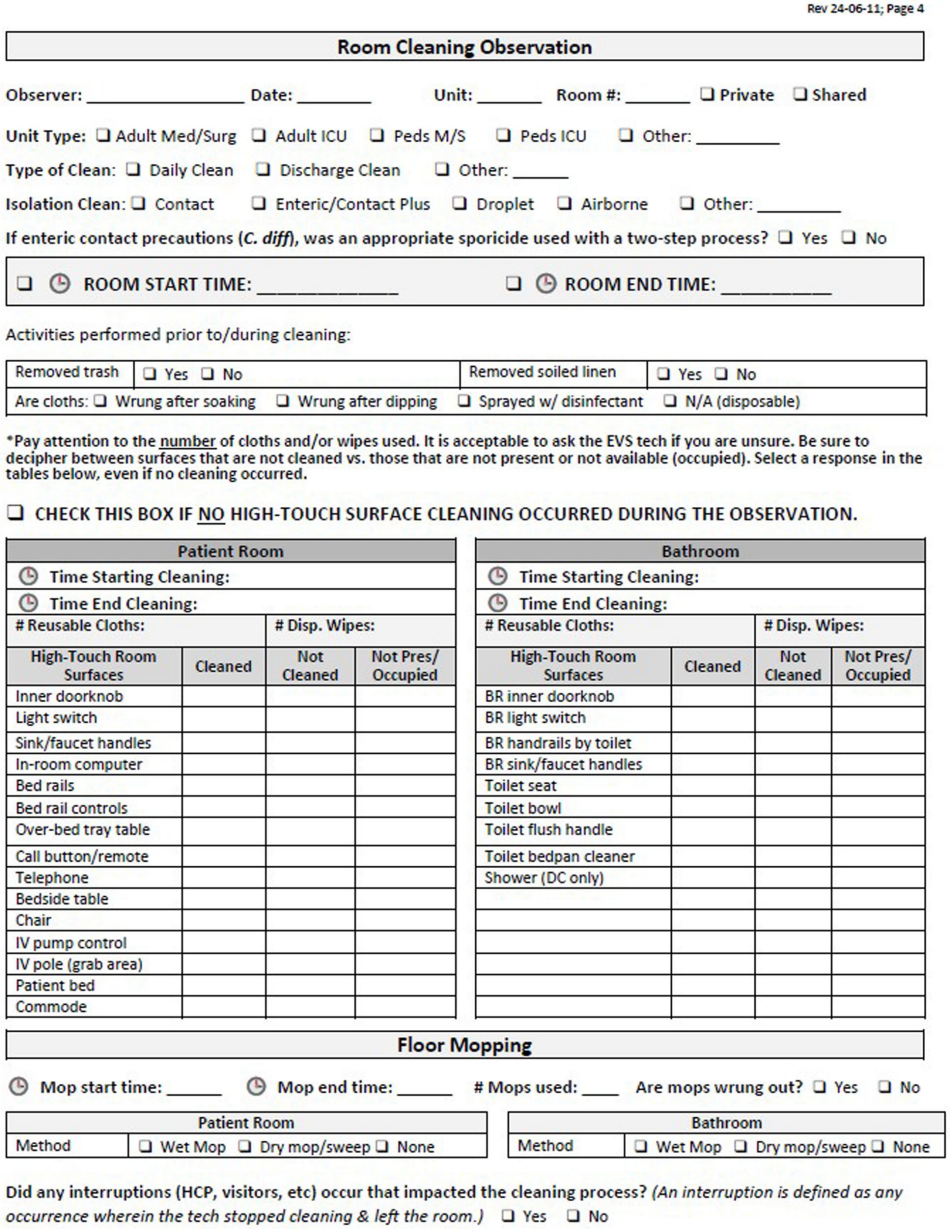

**Figure:**
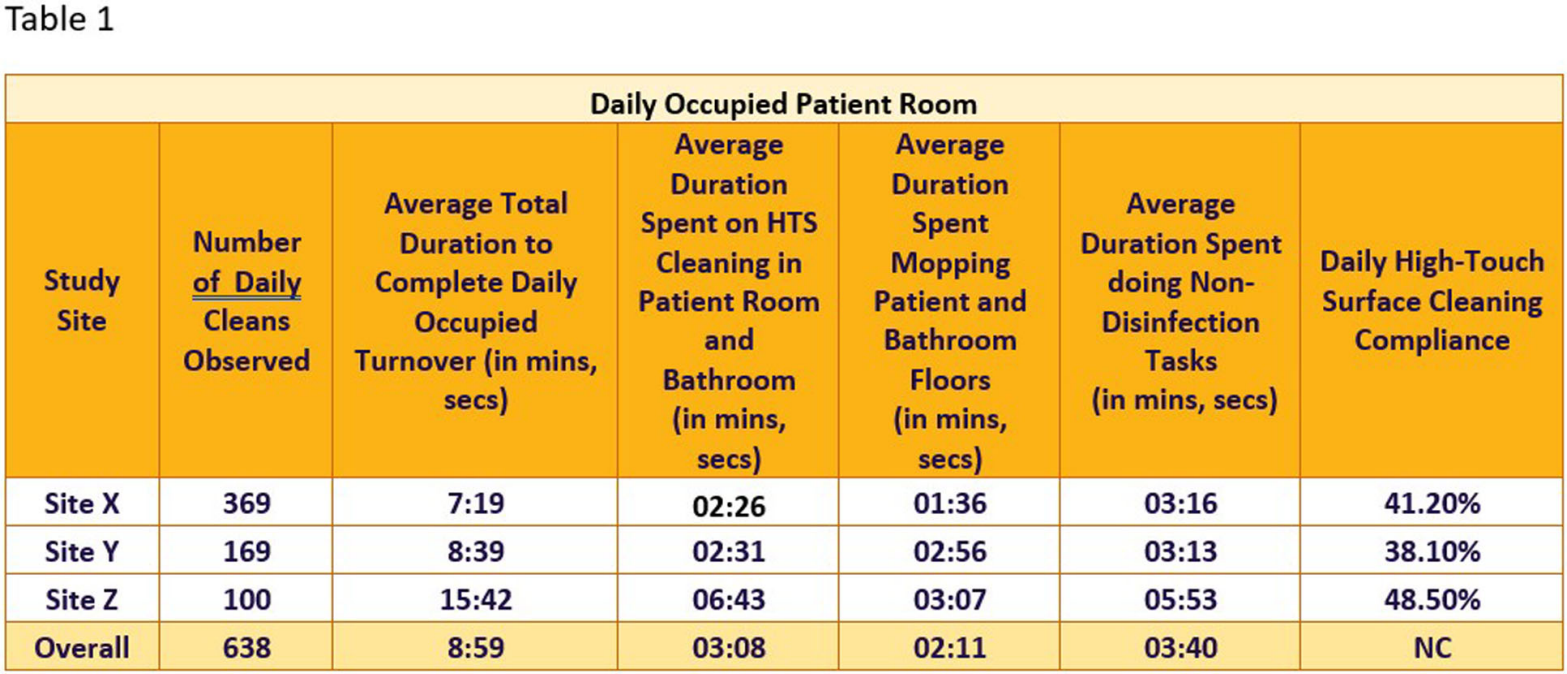

**Figure:**